# Theoretical Study on Redox Potential Control of Iron-Sulfur Cluster by Hydrogen Bonds: A Possibility of Redox Potential Programming

**DOI:** 10.3390/molecules26206129

**Published:** 2021-10-11

**Authors:** Iori Era, Yasutaka Kitagawa, Natsumi Yasuda, Taigo Kamimura, Naoka Amamizu, Hiromasa Sato, Keigo Cho, Mitsutaka Okumura, Masayoshi Nakano

**Affiliations:** 1 Department of Materials Engineering Science, Graduate School of Engineering Science, Osaka University, Toyonaka 560-8531, Japan; iori.era@cheng.es.osaka-u.ac.jp (I.E.); t.kamimura@cheng.es.osaka-u.ac.jp (T.K.); naoka.amamizu@cheng.es.osaka-u.ac.jp (N.A.); hiromasa.sato@cheng.es.osaka-u.ac.jp (H.S.); keigo.cho@cheng.es.osaka-u.ac.jp (K.C.); mnaka@cheng.es.osaka-u.ac.jp (M.N.); 2 Center for Spintronics Research Network (CSRN), Graduate School of Engineering Science, Osaka University, Toyonaka 560-8531, Japan; 3 Quantum Information and Quantum Biology (QIQB) Division, Osaka University, Toyonaka 560-8531, Japan; 4 Research Center for Solar Energy Chemistry (RCSEC), Division of Quantum Photochemical Engineering, Graduate School of Engineering Science, Osaka University, Toyonaka 560-8531, Japan; 5 Department of Chemistry, Graduate School of Science, Osaka University, Toyonaka 560-0043, Japan; nyasuda@chem.sci.osaka-u.ac.jp (N.Y.); ok@chem.sci.osaka-u.ac.jp (M.O.); 6 Innovative Catalysis Science (ICS) Division, Institute for Open and Transdisciplinary Research Initiatives (OTRI), Osaka University, Toyonaka 560-8531, Japan

**Keywords:** [2Fe-2S] ferredoxin, redox potential, hydrogen bond, density functional theory (DFT)

## Abstract

The effect of hydrogen bonds around the active site of *Anabaena* [2Fe-2S] ferredoxin (Fd) on a vertical ionization potential of the reduced state (*IP(red)*) is examined based on the density functional theory (DFT) calculations. The results indicate that a single hydrogen bond increases the relative stability of the reduced state, and shifts *IP(red)* to a reductive side by 0.31–0.33 eV, regardless of the attached sulfur atoms. In addition, the *IP(red)* value can be changed by the number of hydrogen bonds around the active site. The results also suggest that the redox potential of [2Fe-2S] Fd is controlled by the number of hydrogen bonds because *IP(red)* is considered to be a major factor in the redox potential. Furthermore, there is a possibility that the redox potentials of artificial iron-sulfur clusters can be finely controlled by the number of the hydrogen bonds attached to the sulfur atoms of the cluster.

## 1. Introduction

In biosystems, the energy is sometimes transferred by electrons with various potential energies in a stepwise manner [1,2,3]. For this reason, organisms usually have a variety of electron transfer proteins. Iron–sulfur clusters are often found in the active sites of those electron transfer proteins in a wide range of organisms from bacterium to higher organisms [2]. It has been reported that the electron transfer proteins containing such iron-sulfur clusters show a broad range of redox potentials although they have the same clusters in the active sites [3]. For example, [4Fe-4S] ferredoxin (Fd) and high potential iron-sulfur protein (HiPIP) are both the electron transfer proteins that have the same 4Fe-4S clusters in the active sites; nevertheless, their redox potentials are −400 ([Fe_4_S_4_(Cys)_4_]^2−/3−^) and +360 ([Fe_4_S_4_(Cys)_4_]^1−/2−^) mV, respectively [3].

**Scheme 1:**
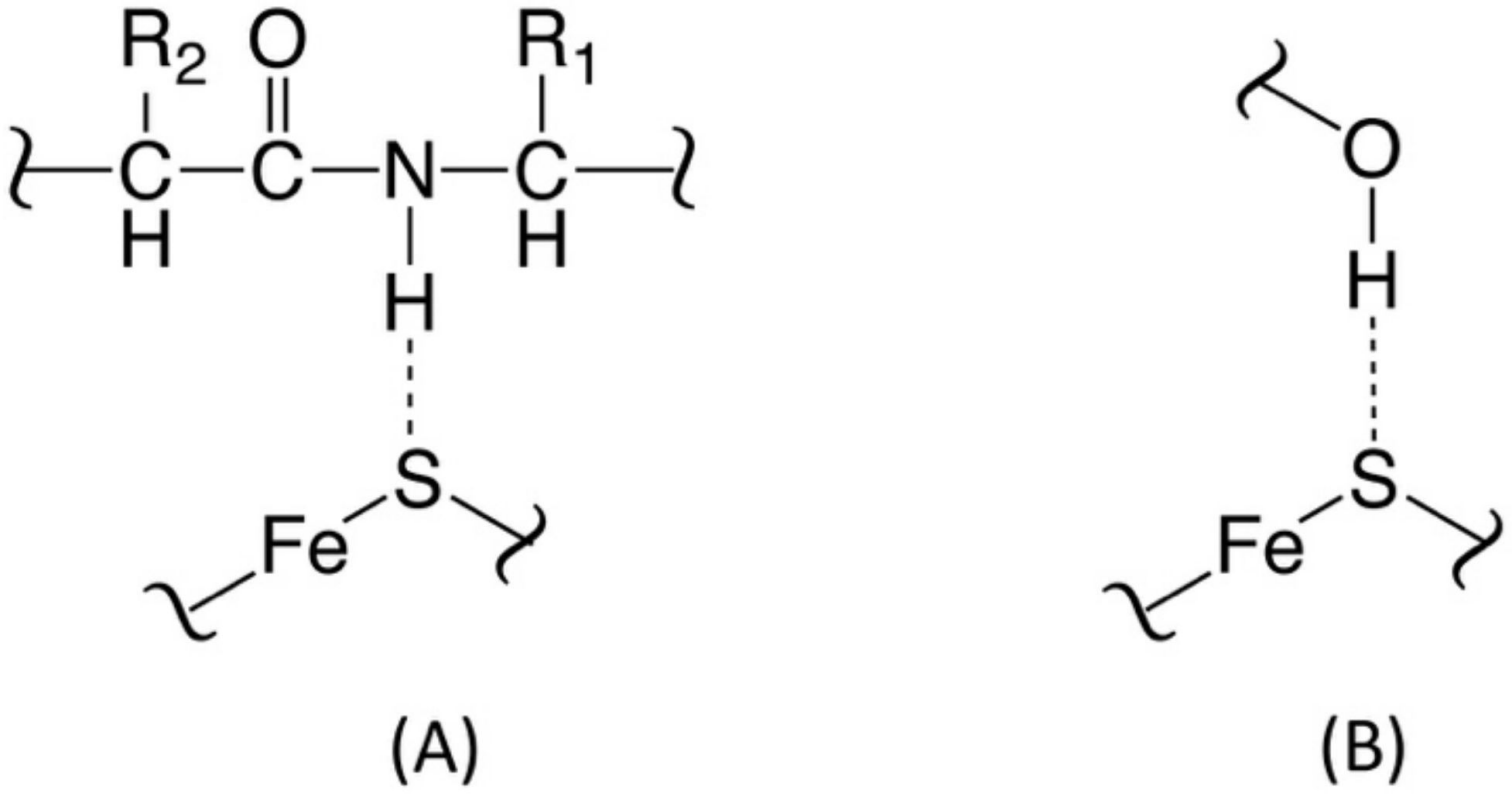
(**A**) The [N-H…S] hydrogen bond between the peptide N-H and sulfur atom of the active site. R_1_ and R_2_ are side chains. (**B**) The [O-H…S] hydrogen bond between the side chain O-H and sulfur atom of the active site.

A mechanism of how they control the redox potentials using the same 4Fe-4S clusters has attracted much attention in both bioscience and material science. Up to now, experimental and theoretical studies have proposed many hypotheses for the mechanism [4,5,6,7,8,9,10,11,12,13,14,15,16,17,18,19,20] such as a distortion of the cubane-type cluster [4], a hydrophobic atmosphere around the active site [6], a protein environment [7,8,9], and hydrogen bonds with solvent waters [10]. On the other hand, a development of X-ray crystallographic analyses has clarified a relationship between the number of hydrogen bonds around the active site and the redox potential [3,11,12]. There have also been some reports that support the importance of the hydrogen bonds by the theoretical calculations. For example, Gámiz-Hernández and co-workers reported that there is a relationship between the hydrogen bonds and the redox potential using optimized [Fe(SCH_2_CH_3_)_4_] structures under the solvent condition [13]. They clarified the importance of the number of hydrogen bonds for the redox potential, however, the effect of each hydrogen bond and its mechanism in the real active sites are still unknown. Usually the iron-sulfur clusters have several [N-H…S] hydrogen bonds between the peptide N-H bonds and sulfur atoms of the cluster as illustrated in Scheme 1. For instance, [4Fe-4S] Fd shows the lower redox potential has eight or nine hydrogen bonds [12], while HiPIP shows higher redox potential has five [6]. In general, the iron-sulfur clusters are negatively charged if coordinating cysteines are included, therefore, a weak positive charge on the hydrogen atom by the charge polarization (–N^δ^^−^–H^δ+^) is expected to stabilize the active site. This hypothesis gives important insight to connect the protein structure and redox potential, however, there has not been direct evidence.

Our group has studied the electronic structures of iron-sulfur complexes by theoretical calculations [17,18,19]. From the result of the 4Fe-4S cluster of HiPIP, we found that the hydrogen bonds around the cluster scarcely change orbital energy gaps, shapes of Kohn–Sham molecular orbitals, and magnetic interactions between Fe ions, although they stabilize orbital energies [19]. In addition, the calculated vertical ionization potential of the reduced state (*IP(red)*) suggests that the hydrogen bonds around the active site stabilize the reduced state by making a positive electrostatic environment. Because the ionization potential is one of the dominant factors of the redox potential of the protein [7,20], the result strongly suggests an importance of the hydrogen bonds for control of the redox potential. A detail of the relationship between the hydrogen bonds and the redox potential, however, has not been made clear yet. The iron–sulfur clusters are often utilized for various catalysts [21,22,23], so that the relationship contributes to a design guideline of such catalysts.

In this study, therefore, we aim to clarify the relationship between the hydrogen bonds around the active site in the iron–sulfur protein and the ionization potential by theoretical calculations in detail. As the first step, we focus on [2Fe-2S] ferredoxin ([2Fe-2S] Fd), which has a simple structure of the active site. In living organisms, one can find various proteins containing the 2Fe-2S clusters that work for electron transfer. For example, the plant-type and adrenodoxin-type Fds that are found in photosynthesis systems and in the metabolic system, respectively, are the well-known 2Fe-2S electron transfer proteins. The [2Fe-2S] Fds show various redox potentials from lower (ca. −450mV [24]) to higher (ca. −270 mV [25]) values, but nevertheless have the same cluster structure. In order to elucidate how the proteins control the redox potentials of the active site, the effect of the hydrogen bonds on the *IP(red)* values is examined by DFT calculations using the model structures of the [2Fe-2S] Fd.

## 2. Computational Models

### 2.1. Calculated Model Structures

**Figure 1:**
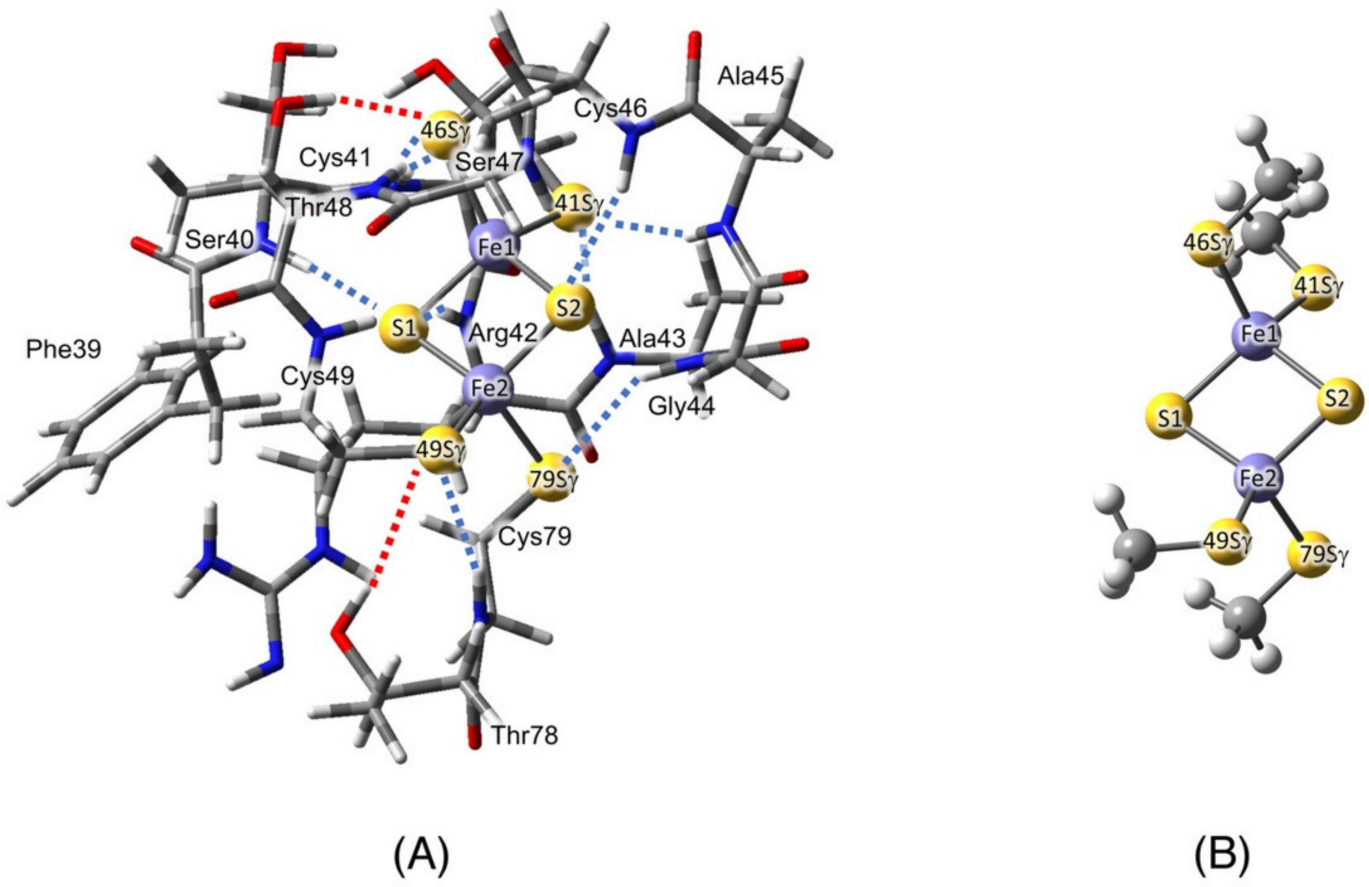
(**A**) Calculated model structure that consists of 13 amino acid residues involving 11 hydrogen bonds (**1_H_**). Blue and red dashed lines indicate nine [N-H…S] and two [O-H…S] hydrogen bonds, respectively. (**B**) Cluster model of the active site where Cys41, Cys46, Cys49, and Cys79 are substituted by methanethiolate ligands (**1_N_**).

A model structure of the [2Fe-2S] Fd is constructed from a result of the X-ray crystallographic analysis for an oxidized form of the plant-type *Anabaena* PCC7119 (PDB ID: 1QT9) [26]. Here, the hydrogen bonds around the 2Fe-2S active site are decided by two definitions as follows: (i) the distance between the hydrogen atom (H) and sulfur (S) (***r***_H–S_) is shorter than 3.2Å; (ii) the angle among proton donor (D), H, and S (∠D-H⋯S) is larger than 139°. Around the active site of *Anabaena* PCC7119, there are 11 hydrogen bonds including nine [N–H⋯S] hydrogen bonds from the peptide N-H bonds as well as two [O–H…S] bonds from side chains of Thr48 and Thr78 as illustrated in Figure 1A and as listed in Table 1. In order to focus on those 11 hydrogen bonds and to reduce a computational cost, we construct the minimal model including 13 amino acid residues (Phe39–Cys49, Thr78 and Cys79) that contain the 11 hydrogen bonds as illustrated in Figure 1A. All those amino acid residues are included in the model as a charge-neutral state. The N and C terminals of the amino acid residues in the model are capped by the hydrogen atoms. The Cartesian coordinates of all hydrogen atoms in the model including the hydrogen bonds are optimized by the DFT calculations, and other atoms are fixed to the X-ray structure.

In order to clarify the effect of the hydrogen bonds, a naked cluster model in which the four cysteines attached to the Fe ions are replaced by methanethiolate (CH_3_S^−^) ligands is also constructed as illustrated in Figure 1B. Here the model structures with/without the 11 hydrogen bonds are abbreviated as **1_H_** and **1_N_**, respectively. Furthermore, the effect of each hydrogen bond is also examined by changing amino acid residues in the model structure as explained below. The Cartesian coordinates of **1_H_** are summarized in Supplementary Materials Table S1.

The charge states of the active site for the reduced and oxidized states are [Fe(II,III)_2_S_2_(Cys)_4_]^3−^ and [Fe(III,III)_2_S_2_(Cys)_4_]^2−^, respectively, including the coordinating Cys^–^ residues [26]. As mentioned above, the attached amino acid residues (oligomer) are the charge neutral states, therefore, net charges of both **1_H_** and **1_N_** are the same. All Fe ions are a high spin state (Fe(II) *s* = 2, Fe(III) *s* = 5/2), and an antiferromagnetic coupling state is assumed as reported in [17] and [18].

### 2.2. Calculation of the Vertical Ionization Potential of the Reduced State (IP(red))

In experiments, a relative stability among several charge states is often discussed by using the redox potential ($E_{redox}$) that is defined by the difference in the free energies between the oxidized (*ox*) and reduced (*red*) states. In recent years, the redox potentials were calculated by the DFT calculations effectively [15,16,27,28]. As discussed later, the quantitative estimation of the redox potential is one of the important issues in the quantum chemistry. Some papers reported the good agreement of the computational results with the experimental ones [27,28], however, it is still a difficult task especially for large systems surrounded by the proteins. To avoid such difficulties, Noodleman and co-workers proposed a simplified method [7],

(1) $$E_{redox}\approx IP\left(red\right)+\Delta E_{solv}+\Delta SHE$$

where $\Delta E_{solv}$ is a difference in solvation-free energies between the *ox* and *red* states. ΔSHE is a correction constant for the standard hydrogen electrode (SHE). We note that ΔSHE is −4.43 V instead of the 4.43 V [7]. $IP\left(red\right)$ is the vertical ionization potential values of the *red* state calculated by

(2) $$IP\left(red\right)=E\left(ox\right)-E\left(red\right)$$

where $E\left(ox\right)$ and $E\left(red\right)$ are calculated total energies of the *ox* and *red* states by the DFT method in the gas phase. It is reported that the *IP(red)* values are a major factor for the $E_{redox}$ in the 4Fe-4S systems, and dominant in some cases such as between [Fe_4_S_4_(SCH_3_)_4_]^0^ and [Fe_4_S_4_(SCH_3_)_4_]^1−^ [7]. For this reason, we discussed the effect of the hydrogen bonds on the redox potential by using *IP(red)* under the assumption that the value is also key factor for the redox potential of the 2Fe-2S systems. The effect of solvation is discussed below.

## 3. Results and Discussion

### 3.1. Electronic Structures of the Oxidized and Reduced States

The calculated electronic structures for the *ox* and *red* states are confirmed by the atomic spin densities on Fe and S atoms as summarized in Table 2. Positive (up) and negative (down) spins on Fe ions indicate the antiferromagnetic spin coupling state. In the case of the *ox* state i.e., [Fe(III,III)_2_S_2_(Cys)_4_]^2−^, the values of the spin densities on Fe1 and Fe2 ions are smaller than the value expected from the high spin Fe(III) (five spins), indicating a small orbital overlap between Fe1 and Fe2 ions as reported in previous studies [17,18]. In addition, the beta spin densities found on 41Sγ and 46Sγ (or the alpha spin densities on 49Sγ and 79Sγ) suggest a partial alpha (or beta) electron transfer from the cysteine residues to the Fe(III) ions, which also decreases the spin densities on Fe(III) ion. In the case of the *red* state i.e., [Fe(II,III)_2_S_2_(Cys)_4_]^3−^, on the other hand, the spin density on Fe1 significantly decreases in comparison with the value of Fe2, indicating that Fe1 is reduced to Fe(II). In the *red* state, the negligible spin densities of 41Sγ and 46Sγ suggest that the partial electron transfer only occurs from cysteines to Fe(III) but not to Fe(II). In addition, the quantitatively equivalent spin density distributions between **1_H_** and **1_N_** indicate that the two models are the same electronic configuration.

### 3.2. Calculated Vertical Ionization Potential (IP(red)) of **1_H_** and **1_N_**

The calculated total energies of the *ox* and *red* states, and *IP(red)* values of **1_H_** and **1_N_** are summarized in Table 3. The calculated *IP(red)* values are negative, meaning that the *ox* state is more stable in comparison with the *red* state on both models. The absolute value of **1_H_** (1.65 eV) is, however, significantly smaller than the value of **1_N_** (5.06 eV). The difference in *IP(red)*, which is about 3.4 eV, indicates that the *red* state is drastically stabilized by the amino acid residues around the active site compared to the *ox* state. In other words, the existence of the surrounding amino acids increases the relative stability of the *red* state. As mentioned above, the attached amino acid residues (oligomer) in **1_H_** are neutral charge, so that the net charge of the system is equivalent between **1_H_** and **1_N_**. In addition, the electronic configurations of **1_H_** and **1_N_** are also the same as shown in Table 2, therefore, the difference in *IP(red)* does not originate in the change of the charge or spin states, but in the effect of the surrounding amino acid residues. The results suggest two possibilities of mechanisms for change in *IP(red)* by the amino acid residues: (1) a local interaction by the hydrogen bonds, and (2) a whole electrostatic potential by residues. If the effect originates in the hydrogen bonds, *IP(red)* must be changed by single or several amino acids. In order to clarify the effect of the hydrogen bonds on *IP(red)* in detail, therefore, the effect of each hydrogen bond on *IP(red)* is separately examined.

### 3.3. Effect of [N-H…S] Hydrogen Bond from the Peptide on IP(red)

**Figure 2:**
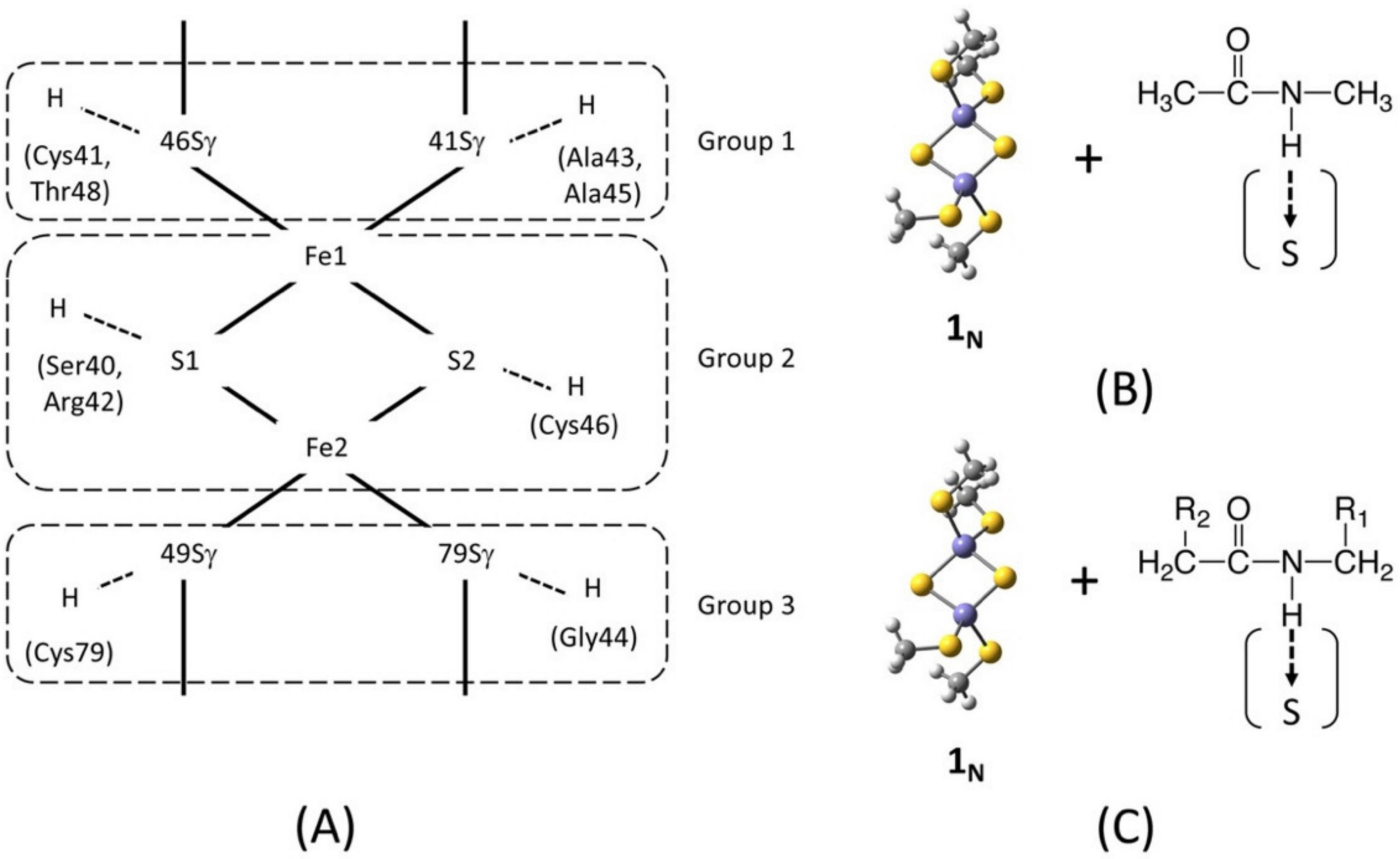
(**A**) Illustration of three groups of hydrogen bonds classified by the attached sulfurs. (**B**) The peptide model that consists of **1_N_** and peptide moiety without the side chains. (**C**) The amino acid model that consists of **1_N_** and peptide moiety with the side chains (R_1_ and R_2_). For both peptide and amino acid models, the proton donor is defined as the amino acid residue providing N-H bond.

As mentioned above, there are 11 hydrogen bonds including nine [N-H…S] from the peptide N-H, and two [O-H…S] from the side chains in **1_H_**. First, we confirm the effect of the [N-H…S] hydrogen bonds. As listed in Table 1, those hydrogen bonds are classified into three groups (Group 1–3) based on the attached sulfur atoms (Figure 2A). The hydrogen bonds in the group 1 and group 3 are bound to sulfur atoms of cysteines coordinating to Fe1 and Fe2, respectively, while those in group 2 are bound to bridging (inorganic) sulfur atoms. For each [N-H…S] hydrogen bond, we construct simple models, namely the peptide model, that consists of **1_N_** and a peptide bond moiety as illustrated in Figure 2B. In the model, both N and C terminals are capped by hydrogen atoms. Only the geometry of the hydrogen atoms is optimized, but other atoms are fixed to **1_H_**. Some examples of the model are depicted in Supplementary Materials Figure S1. The calculated total energies and *IP(red)* values are summarized in Table 4. The calculated *IP(red)* values are in a range −4.65 to −4.72 eV, when a single hydrogen bond is attached to **1_N_**. Those values are smaller than the value of **1_N_** by 0.3–0.4 eV, indicating that the single peptide N-H bond stabilizes the *red* state in comparison with the *ox* state. On the other hand, there are no *special* hydrogen bonds that dominantly contribute to *IP(red)*. In other words, all hydrogen bonds seem to contribute to *IP(red)* equivalently.

**Figure 3:**
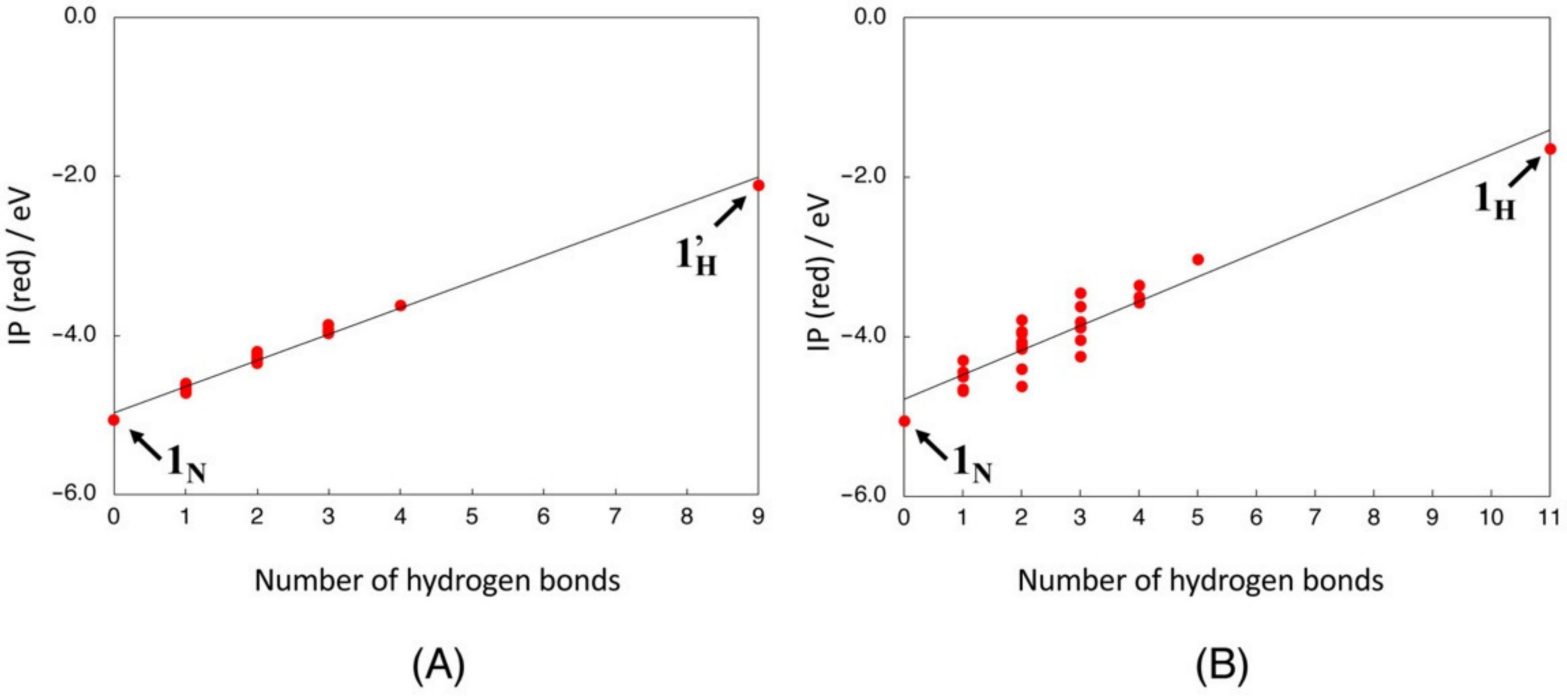
The relationships between the number of hydrogen bonds and calculated *IP(red)* values. (**A**) The peptide models. (**B**) The amino acid models.

In order to confirm a synergistic effect among hydrogen bonds on *IP(red)*, a combination of hydrogen bonds within each group are examined as summarized in Table 4. For example, there are four amino acid residues that have [N-H…S] hydrogen bonds in group 1 (Cys41, Ala43, Ala45, Thr48), so there are 11 combinations among the choices of the residues i.e., (Cys41, Ala43), (Cys41, Ala45), …, (Cys41, Ala43, Ala45, Thr48). For the comparison, a model where all side chains of **1_H_** are substituted by hydrogen atoms is also constructed (**1_H_’**). Because **1_H_’** consists of the main chain, it only includes nine [N-H…S] hydrogen bonds. The calculated total energies of the *ox* and *red* states and the *IP(red)* values are summarized in Table 4. With an increase in the amino acid residues of the computational models, i.e., an increase of the number of hydrogen bonds, the absolute values of the calculated *IP(red)* become smaller. In Figure 3A, those calculated *IP(red)* values are plotted against the number of the hydrogen bonds. The result clearly indicates that the *IP(red)* values are in direct proportion to the number of the hydrogen bonds. It shows that the number of the hydrogen bonds contributes to *IP(red)*, but the synergistic effect is not found. The slope of the plot is 0.33, indicating that a single hydrogen bond increases the relative stability of the *red* state and shifts *IP(red)* to the reductive side by 0.33 eV.

### 3.4. Effect of [O-H…S] Hydrogen Bond from the Side Chains on IP(red)

The effect of the [O-H…S] hydrogen bonds is also confirmed by using the models including the side chains. The models including the side chains, namely the amino acid model, are constructed by using **1_N_** and peptide bond moiety with the side chains as illustrated in Figure 2C. In the model, both N and C terminals are capped by hydrogen atoms. The geometry of the hydrogen atoms is optimized, but other atoms are fixed to **1_H_**. Some examples of the models are depicted in Supplementary Materials Figure S1. According to the strategy used for the peptide model, the *IP(red)* values are calculated by using the combination of the amino acid residues summarized in Table 5. Unlike the peptide model, Thr48 provides both [N-H…S] and [O-H…S] hydrogen bonds in the amino acid model. On the other hand, Thr78, which only provides [O-H…S] hydrogen bond from the side chain, is included in the model of Cys79 as [Cys79 + Thr78], as illustrated in Supplementary Materials Figure S1.

The calculated total energies of the *ox* and *red* states and *IP(red)* values are summarized in Table 5. The calculated *IP(red)* values are weakly perturbed by the side chains in comparison with the peptide models. The plot in Figure 3B, however, keeps a proportional relationship between the number of the hydrogen bonds and the *IP(red)* values even with the side chains. The slope (0.31) expresses that a single hydrogen bond shifts *IP(red)* to the reductive side by 0.31 eV, which is almost equivalent to the value of the peptide model. In contrast to the peptide model, the amino acid model shows the larger dispersion. The difference in the *IP(red*) values between the amino acid and peptide models is significant in Ala43, Thr48, Ser40, and Arg42. Those amino acids involve side chains that contribute the hydrogen bonds except for Ala43. In addition to the difference in a strength of the hydrogen bond between [N-H…S] and [O-H…S] [13], a part of the dispersion of the *IP(red*) values is considered to originate in the inter-amino acid interaction, although further analysis must be needed. On the other hand, a distribution of the H-S distance (***r***) and N-H-S angle (*θ*) does not affect the dispersion of *IP(red)* so much as shown in Supplementary Materials Figure S2.

### 3.5. Possible Mechanism for the Effect of Hydrogen Bond on IP(red)

**Figure 4:**
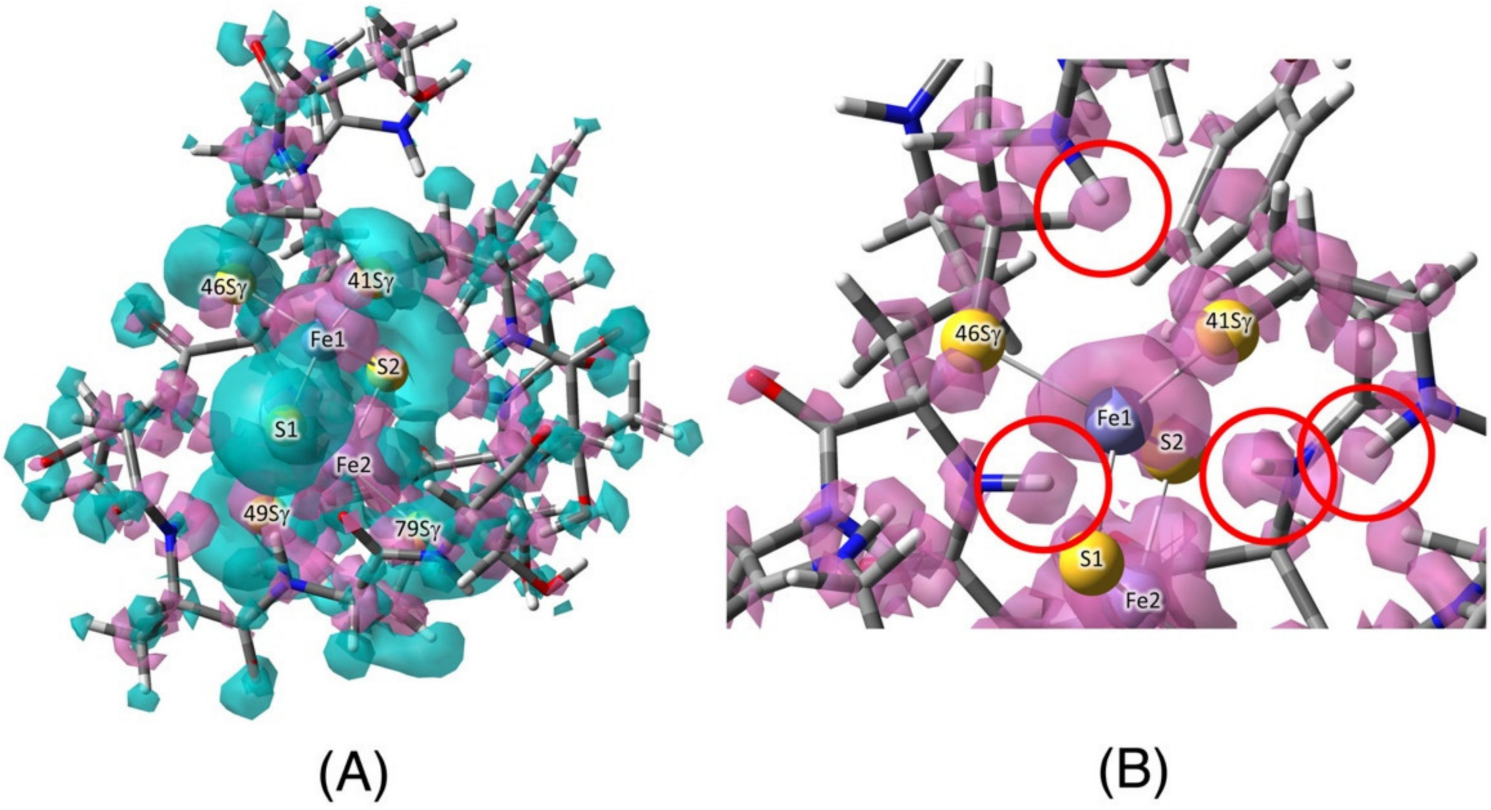
(**A**) An electron density difference map (Δρ) between the oxidized (*ox*) and reduced (*red*) states (ρ(*ox*)–ρ(*red*)). Purple and blue colors show positive and negative distributions, respectively. (**B**) Enlarged view around active sites, which shows only the purple distribution. The areas indicated by circles show positions of the hydrogen atoms concerned with the hydrogen bonds.

In order to explain why the hydrogen bonds further stabilize the *red* state and shift *IP(red)* to the reductive side, an electron density difference map (Δρ) between the *ox* and *red* states (ρ(*ox*)–ρ(*red*)) is depicted in Figure 4A, where ρ is the electron density distribution. The blue (negative) area is largely distributed especially on the active site because the *red* state has one more electron than the *ox* state. On the contrary, the hydrogen atoms concerning the hydrogen bonds around the active site are found to be colored by purple (positive). Figure 4B that focuses on the positive distribution around active sites clearly indicates that the hydrogen atoms concerning the hydrogen bonds become positive, indicating that the electron densities at those hydrogen positions are decreased in the reduced state. This suggests that the hydrogen atom concerned with the hydrogen bonds shows a stronger charge polarization (N^δ−^–H^δ+^) in the reduced state. As a result, the local positive electrostatic field by the hydrogen bonds is enhanced especially for the *red* state. The hydrogen bonds, therefore, are considered to shift *IP(red)* to the reductive side.

## 4. Materials and Methods

All calculations are performed by using the spin-unrestricted hybrid DFT method with the B3LYP functional set on the Gaussian program package [29]. Two different levels of basis sets i.e., **BS-I** (Fe, C, H: 6-31G, N, O: 6-31G* and S: 6-31+G*) and **BS-II** (Fe, C, N, O, H: 6-31G* and S: 6-31+G*) are used for the geometry optimization of hydrogen atoms, and following single-point energy calculations, respectively. Those calculations are carried out in the gas phase condition, unless otherwise specifically noted.

## 5. Conclusions

In this paper, the effect of the hydrogen bonds around the active site of *Anabaena* [2Fe2S] Fd on *IP(red)* is examined based on the DFT calculations. The results indicate that a single [N-H…S] or [O-H…S] hydrogen bond can shift *IP(red)* to the reductive side by 0.31–0.33 eV, regardless of the attached sulfur atoms. In addition, the value can be changed by the number of hydrogen bonds. Those results strongly suggest that the difference in *IP(red)* between the **1_N_** and **1_H_** (3.4 eV) originates in a summation of the local effects of the 11 hydrogen bonds included in the **1_H_** model rather than the effect by the whole amino acid residues (oligomer) included in the model. The results also suggest that the redox potential of [2Fe-2S] Fd can be accurately controlled by the number of hydrogen bonds. The information gives us some useful insights for mechanisms of the electron transfer as well as the control of the redox potential. For example, the X-ray structural analysis has revealed that the reduced structure of this protein (PDB ID: 1CZP) has one additional [N-H…S] hydrogen bond from Cys46 around the active site (12 hydrogen bonds in total) [26]. In other words, there is some possibility of controlling the electron transfer (giving and receiving) by the change in the number of the hydrogen bonds around the active site. Furthermore, the Fe1 site, which has more hydrogen bonds, is expected to be easily reduced, as supported by the calculated result in Table 1.

The importance of the hydrogen bonds around the active site is explained in this study, however, the obtained value (−1.65 eV is considered to be −1.65 V in the case of the one-electron transfer) is different from the experimental values even if ΔSHE is considered. The result suggests the strong contribution of environmental effect from the outer region i.e., the surrounding protein or water molecules. In fact, it is reported that $\Delta E_{solv}$ becomes more important especially for more negatively charged [Fe_4_S_4_(SCH_3_)_4_] model systems [7]. As mentioned above, in addition, Gámiz-Hernández et al. estimated a change in the redox potential by the number of hydrogen bonds using the optimized [Fe(SCH_2_CH_3_)_4_] model clusters under the solvent condition. Our result is a few times larger than their conclusion (a change is 0.064–0.095 eV per hydrogen bond) [13]. In order to evaluate the importance of the surrounding environment, we calculate *IP(red)* of **1_H_** and **1_N_** under a solvent (water) condition with the polarizable continuum model (PCM) using the integral equation formalism variant (IEFPCM). The calculated total energies and *IP(red)* are summarized in Table 5. The calculated *IP(red)* values (3.32 and 2.89 eV for **1_H_** and **1_N_**, respectively) is drastically changed by the solvent water. It is reported that the solvent effect considered by the IEFPCM sometimes overestimates the electrostatic effect [19], however the result strongly suggests the importance of the environmental effect from the outer region on the redox potential, too. In addition, the differences between **1_H_** and **1_N_** (0.43 eV) are decreased a lot by the solvent effect, indicating that the dielectric field of the polar solvent stabilized the *red* state as a substitute for the positive electrostatic field from the hydrogen bonds in **1_N_**. Conversely, the effect of hydrogen bonds around the active sites is expected to stand out especially in a hydrophobic atmosphere condition such as HiPIP. In fact, the difference between **1_H_** and **1_N_** (3.4 eV) can be also said to be almost comparable to the difference between the gas phase and solvent of **1_H_** (4.97 eV), suggesting the strong contribution of the local hydrogen bonds around the active site.

Considering the functionality of the protein, in addition, the electron transfer (giving and receiving) must be controlled accurately. From the above results, a local rearrangement of the hydrogen bonds is considered to be suitable for the accurate control of the redox for the electron transfer, while the surrounding protein and solvents are suited to shift the redox potential significantly. In order to clarify roles of the hydrogen bonds and protein, ONIOM calculations including a whole protein and water molecules is necessary, but we focus on the effect of the hydrogen bonds and do not discuss further about the effect from the outer region here. In addition to the consideration of the surrounding protein, the quantitative estimation of the redox potential is also required for the further elucidation of the mechanism. There are many reports about the theoretical calculation of the redox potentials [15,16,27,28,30]. Those approaches must also be considered for the quantitative analyses and further discussions.

There have been, on the other hand, many reports about the synthesized iron–sulfur cluster complexes that aim to add catalytic functionality and so on [22,23]. The above results suggest that the redox potentials of such artificial iron-sulfur clusters can be finely controlled by the number of the hydrogen bonds attached to the sulfur atoms of the clusters. The tune or control of the redox potential by the number of hydrogen bonds can be an effective designing guideline for the iron-sulfur clusters, suggesting a possibility of potential redox programming by the surrounding ligands.

## Figures and Tables

**Table 1:** Structural parameters about the 11 hydrogen bonds around the active site included in the model structure (**1_H_**). The proton donor (D) is the amino acid residue that provides an N-H (or O-H) bond. The D-S distance is the distance between N and S (or O and S). The geometry of hydrogen atoms is optimized but the position of other atoms is fixed to the X-ray structure. Groups of the hydrogen bonds are classified by attaching sulfur atoms as shown in Figure 2A.

Type of Hydrogen Bond	Proton Donor (D)	Sulfur (S)	D-S Distance/Å	*r*_H–S_/Å	∠D-H⋯S/deg	Group
[N-H…S]	Cys41	46Sγ	3.94	2.93	172.0	Group 1
	Ala43	41Sγ	3.28	2.29	154.1	
	Ala45	41Sγ	3.39	2.46	146.6	
	Thr48	46Sγ	3.40	2.40	165.6	
	Ser40	S1	3.28	2.26	176.4	Group 2
	Arg42	S1	3.29	2.32	160.7	
	Cys46	S2	3.35	2.49	142.4	
	Gly44	79Sγ	3.54	2.63	148.8	Group 3
	Cys79	49Sγ	3.55	2.56	163.9	
[O-H…S] ^a)^	Thr48	46Sγ	3.49	2.59	153.8	Group 1
	Thr78 ^b)^	49Sγ	3.95	3.16	139.0	Group 3

^a)^ The O-H in the side chain provides the [O-H…S] type hydrogen bond; ^b)^ Thr78 does not provide the [N-H…S] hydrogen bond but the O-H in the side chain provides the [O-H…S] hydrogen bond.

**Table 2:** Calculated atomic spin densities of oxidized (*ox*) and reduced (*red*) states of **1_H_** and **1_N_** ^a)^.

States	Atoms ^b)^							
	Fe1	Fe2	S1	S2	41Sγ	46Sγ	49Sγ	79Sγ
*ox*	−3.82 (−3.75)	3.75 (3.72)	−0.06 (0.02)	−0.07 (0.01)	−0.16 (−0.22)	−0.19 (−0.25)	0.26 (0.23)	0.28 (0.24)
*red*	−3.68 (−3.52)	3.79 (3.72)	0.17 (0.28)	0.29 (0.31)	−0.03 (−0.07)	−0.03 (−0.10)	0.22 (0.19)	0.21 (0.18)

^a)^ Values of **1_N_** are in parentheses; ^b)^ 41Sγ and 46Sγ coordinate to Fe1, and 49Sγ and 79Sγ coordinate to Fe2. See Figure 1A.

**Table 3:** Calculated total energies of the *ox* and *red* states, and *IP(red)* values for models **1_H_** and **1_N_**.

Model	Total Energy/Hartree		*IP(red)*/eV
	*ox* State	*red* State	
**1_N_**	−5076.2451	−5076.0590	−5.06
**1_H_**	−8648.4040	−8648.3435	−1.65
**1_H_’** ^a)^	−7440.6109	−7440.5334	−2.11

^a)^ A model where side chains are substituted by hydrogen atoms.

**Table 4:** Calculated total energies of the *ox* and *red* states and the *IP(red)* values of the peptide bond models.

Number of Hydrogen Bonds	Donor	Group	Total Energy/Hartree		*IP(red)*/eV
			*ox* State	*red* State	
1	Cys41	Group 1	−5323.5846	−5323.4139	−4.65
	Ala43		−5324.7894	−5324.6185	−4.65
	Ala45		−5324.7805	−5324.6077	−4.70
	Thr48		−5324.7866	−5324.6139	−4.70
	Ser40	Group 2	−5324.7890	−5324.6200	−4.60
	Arg42		−5323.5841	−5323.4138	−4.63
	Cys46		−5323.5845	−5323.4120	−4.69
	Gly44	Group 3	−5324.7886	−5324.6168	−4.68
	Cys79		−5323.5894	−5323.4159	−4.72
2	Cys41, Ala43	Group 1	−5572.1223	−5571.9652	−4.28
	Cys41, Ala45		−5572.1153	−5571.9569	−4.31
	Cys41, Thr48		−5572.1213	−5571.9633	−4.30
	Ala43, Ala45		−5573.3182	−5573.1597	−4.31
	Ala43, Thr48		−5573.3266	−5573.1689	−4.29
	Ala45, Thr48		−5573.3180	−5573.1584	−4.34
	Ser40, Arg42	Group 2	−5572.1189	−5571.9645	−4.20
	Ser40, Cys46		−5572.1239	−5571.9679	−4.25
	Ser40, Cys46		−5570.9203	−5570.7632	−4.27
	Gly44, Cys79	Group 3	−5572.1282	−5571.9683	−4.35
3	Cys41, Ala43, Ala45	Group 1	−5820.6497	−5820.5042	−3.96
	Cys41, Ala43, Thr48		−5820.6581	−5820.5136	−3.93
	Cys41, Ala45, Thr48		−5820.6508	−5820.5048	−3.97
	Ala43, Ala45, Thr48		−5821.8544	−5821.7086	−3.97
	Ser40, Arg42, Cys46	Group 2	−5819.4560	−5819.3141	−3.86
4	Cys41, Ala43, Ala45, Thr48	Group 1	−6069.1848	−6069.0517	−3.62
9	**1_H_’**		−7440.6109	−7440.5334	−2.11
0	**1_N_**		−5076.2451	−5076.0590	−5.06

**Table 5:** Calculated total energies of the *ox* and *red* states and the *IP(red*) values of the amino acid models. The result under the solvent (water) effect for **1_H_** and **1_N_** is written in square parentheses.

Number of Hydrogen Bonds	Donor	Group	Total Energy/Hartree		*IP(red)*/eV
			*ox* State	*red* State	
1	Cys41	Group 1	−5438.1031	−5437.9377	−4.50
1	Ala43		−5686.1898	−5686.0264	−4.45
1	Ala45		−5364.0965	−5363.9243	−4.68
2	Thr48 ^a)^		−5593.1554	−5592.9936	−4.40
1	Ser40	Group 2	−5709.6638	−5709.5060	−4.29
1	Arg42		−5645.6609	−5645.4976	−4.44
1	Cys46		−5362.8987	−5362.7272	−4.67
1	Gly44	Group 3	−5364.1027	−5363.9314	−4.66
2	[Cys79 + Thr78] ^b)^		−5477.4287	−5477.2587	−4.62
2	Cys41, Ala43	Group 1	−6048.0384	−6047.8936	−3.94
2	Cys41, Ala45		−5725.9494	−5725.7968	−4.15
3	Cys41, Thr48 ^a)^		−5955.0059	−5954.8629	−3.89
2	Ala43, Ala45		−5974.0329	−5973.8819	−4.11
3	Ala43, Thr48 ^a)^		−6203.0941	−6202.9540	−3.81
3	Ala45, Thr48 ^a)^		−5881.0021	−5880.8534	−4.05
2	Ser40, Arg42	Group 2	−6279.0694	−6278.9301	−3.79
2	Ser40, Cys46		−5996.3130	−5996.1675	−3.96
2	Ser40, Cys46		−5932.3110	−5932.1615	−4.07
3	Gly44, [Cys79 + Thr78] ^b)^	Group 3	−5765.2810	−5765.1250	−4.24
3	Cys41, Ala43, Ala45	Group 1	−6335.8810	−6335.7479	−3.62
4	Cys41, Ala43, Thr48 ^a)^		−6564.9397	−6564.8166	−3.35
4	Cys41, Ala45, Thr48 ^a)^		−6242.8505	−6242.7194	−3.57
4	Ala43, Ala45, Thr48 ^a)^		−6490.9357	−6490.8073	−3.50
3	Ser40, Arg42, Cys46	Group 2	−6565.7175	−6565.5907	−3.45
5	Cys41, Ala43, Ala45, Thr48 ^a)^	Group 1	−6852.7799	−6852.6688	−3.03
11	1_H_		−8648.4040 [−8648.6202]	−8648.3435 [−8648.7427]	−1.65 [3.32]
0	1_N_		−5076.2451 [−5076.5020]	−5076.0590 [−5076.6082]	−5.06 [2.89]

^a)^ [O-H…S] type hydrogen bond from the side chain of Thr48 is included; ^b)^ [O-H…S] type hydrogen bond from the side chain of adjacent Thr78 is included.

## Data Availability

Not applicable.

## Supplementary Materials

The following are available online. Table S1: Atomic Cartesian coordinates of **1_H_**, Figure S1: Illustration of the peptide and amino acid models. Figure S2: A change in *IP(red)* values by the structural change of the hydrogen bond.
