# Supplementary material for: Theoretical Study on Redox Potential Control of Iron-Sulfur Cluster by Hydrogen Bonds: A Possibility of Redox Potential Programming
Source: Molecules. 2021 Oct 11;26(20):6129. doi: 10.3390/molecules26206129 (PMC8538827; doi:10.3390/molecules26206129)
Supplement: Supplementary file 1 [file molecules-26-06129-s001.zip › molecules-1387346-supplementary.pdf]

Supplementary Materials for

Theoretical study on redox potential control of Iron-Sulfur cluster by hydrogen-bonds: A possibility of redox potential programming

Iori Era [1], Yasutaka Kitagawa [1,2,3,4,*], Natsumi Yasuda [5], Taigo Kamimura [1], Naoka Amamizu [1], Hiromasa Sato [1], Keigo Cho[1], Mitsutaka Okumura [5,6], Masayoshi Nakano [1,2,3,4,6]

[1] Department of Materials Engineering Science, Graduate School of Engineering Science, Osaka University, Toyonaka, Osaka 560-8531, Japan.
[2] Center for Spintronics Research Network (CSRN), Graduate School of Engineering Science, Osaka University, Toyonaka, Osaka 560-8531, Japan.
[3] Quantum Information and Quantum Biology (QIQB) Division, Osaka University, Toyonaka, Osaka 560-8531, Japan.
[4] Research Center for Solar Energy Chemistry (RCSEC), Division of Quantum Photochemical Engineering, Graduate School of Engineering Science, Osaka University, Toyonaka, Osaka 560-8531, Japan.
[5] Department of Chemistry, Graduate School of Science, Osaka University, Toyonaka, Osaka 560-0043, Japan.
[6] Innovative Catalysis Science (ICS) Division, Institute for Open and Transdisciplinary Research Initiatives (OTRI), Osaka University, Toyonaka, Osaka 560-8531, Japan.
* Correspondence: kitagawa@cheng.es.osaka-u.ac.jp

Table S1. Atomic Cartesian coordinates of $\mathbf{1_H}$ (*xyz* format). Unit is Å

161

Fe	1.28899409	22.27300707	30.06301561
Fe	4.01395093	21.94999706	30.04495485
S	2.50200057	20.53200476	29.23400669
S	2.86500066	23.56100539	30.98900711
C	1.13860819	16.76369875	28.63830440
O	0.54797514	15.73999663	28.37709346
C	3.49703380	17.14961994	27.82268134
C	3.26192275	16.38862279	26.55740705
C	2.34964254	16.79563185	25.59223485
C	3.90302589	15.22394452	26.27309800
C	2.10570349	16.12081767	24.42118957
C	3.71233184	14.49827333	25.06576974
C	2.83506965	14.96809150	24.13594255
N	0.61438029	17.96031959	28.47019358
C	-0.70986932	18.20058252	27.96795531
C	-0.73172119	19.41129953	27.00632325
O	-0.75808518	19.24200843	25.77200790
C	-1.74699737	18.47491526	29.09003767
O	-3.05300468	18.59195942	28.56900753
C	-0.73590513	21.83403395	26.72097913
C	0.47398013	22.15267512	25.86172180
O	0.33201408	22.78012419	24.79607371
C	-1.11600826	23.04899026	27.60903634
S	0.23601507	23.76000641	28.59798554
N	1.65508648	21.77215879	26.30238331
C	2.93077937	22.07504630	25.70575358
C	3.19272980	23.54903540	25.53578286
O	3.94410900	23.94500053	24.62609271
C	3.15720073	21.34397783	24.42304058
C	3.13696273	19.85498656	24.47303058
C	3.64995686	19.28403346	23.15095726
N	5.08900417	19.18899439	22.87403526
C	5.88000132	18.14100113	23.11399423
N	5.39000324	17.04700792	23.69201639
N	7.15000063	18.08300114	22.80500023
N	2.54910946	24.38000958	26.35739922
C	2.57428477	25.80613396	26.19952766
C	2.89317286	26.59193994	27.47500628
O	2.79091647	27.82499834	27.49203737
C	1.24292724	26.29396500	25.59513299

N	3.34459660	25.92494398	28.51914258
C	3.69420397	26.60499591	29.77102889
C	2.48683654	26.99750530	30.60854486
O	2.71204663	27.81682631	31.49516224
N	1.32801718	26.46552892	30.31827188
C	0.05104207	26.96234534	30.90210716
C	-0.67696420	25.89894487	31.68994842
O	-1.86000730	26.02299026	31.92198079
C	-0.83400422	27.56294829	29.80997782
C	-0.62095644	23.77800558	32.97703004
C	0.47414610	23.22279731	33.87077422
O	1.56796228	23.78607056	33.92708900
C	-1.37902443	22.77302030	32.11900644
S	-0.36199992	21.42000393	31.38101141
N	0.16877968	22.17205582	34.58722942
C	1.18925693	21.57616922	35.46197878
C	1.60224815	20.16910152	35.03980323
O	2.36489072	19.51295157	35.73308014
C	0.64289794	21.60791662	36.89095299
O	-0.52099398	20.85899789	37.01702920
N	1.08861529	19.66788038	33.90632805
C	1.26022456	18.26699697	33.57328903
C	2.72996870	17.86520850	33.42755536
O	3.11208769	16.78618477	33.83240765
C	0.45594486	17.88902323	32.31304045
O	-0.93500321	18.18702093	32.55797268
C	0.52997720	16.40199282	32.01605321
C	5.35559127	19.16871839	31.31573109
S	5.42777623	20.95403775	31.54282322
H	4.55295975	17.05073058	28.11116650
H	3.33412215	18.21887697	27.64505072
H	1.75895446	17.68538529	25.78872598
H	4.61863198	14.82188067	26.98998014
H	1.36447978	16.49888375	23.72528249
H	4.28227385	13.58920661	24.90034803
H	2.68288250	14.44234877	23.19757365
H	-1.76664308	17.62036486	29.77290959
H	-1.45568579	19.34734857	29.69059977
H	-3.09257698	19.40944545	28.04640806
H	-1.02716901	17.32886524	27.39556333
H	1.17313971	18.77031517	28.74036629
H	4.12558590	21.69059739	24.03972442
H	2.41530489	21.69436529	23.69273078

H	3.74063060	19.46864457	25.30773477
H	2.11435328	19.49221084	24.62802792
H	3.24269692	18.27558176	23.00841654
H	3.21836729	19.88354752	22.33736354
H	5.56916466	19.98769178	22.48459669
H	4.53634244	17.09865112	24.23579285
H	6.06872683	16.44428954	24.12648975
H	7.53292895	17.18469910	23.09006912
H	3.66487671	21.72359212	26.44654215
H	1.70237599	21.28539203	27.19604620
H	1.26473846	27.37921943	25.46563083
H	0.40786331	26.04050651	26.25851621
H	1.07644185	25.80234806	24.63278691
H	3.38260142	26.01816348	25.48944122
H	1.85011070	24.00086265	26.99677933
H	4.33557613	25.94337248	30.35566836
H	4.22616353	27.53179149	29.54855134
H	3.55825107	24.93793094	28.42001339
H	-1.77691765	27.90900772	30.24057353
H	-0.31465244	28.39801888	29.33108437
H	-1.06296331	26.81149897	29.04601187
H	0.36212575	27.74407476	31.60784858
H	1.25907478	25.83701886	29.52580143
H	1.42852527	21.26894012	37.58188316
H	0.38845547	22.64278189	37.14038322
H	-0.27552642	19.92135327	36.94978506
H	2.06416838	22.22781866	35.38502667
H	-0.76455206	21.78354007	34.61121427
H	0.82199670	18.47323406	31.45813772
H	0.08362774	15.84264104	32.84601348
H	-0.02941930	16.17039083	31.10678387
H	1.55858052	16.05469432	31.89692996
H	-1.09764666	19.08356091	32.21205983
H	0.88639541	17.65668340	34.40294254
H	0.55582298	20.27052045	33.27929842
H	-1.54413625	21.67616181	25.99669089
H	-1.95349260	22.76425538	28.25779627
H	-1.46299192	23.84616192	26.94500626
H	-1.37269352	24.27415545	33.61347655
H	-2.15345138	22.27169889	32.71445605
H	-1.88615267	23.32218870	31.32308502
H	6.32783069	18.79999117	30.97288499
H	4.63373258	18.91610684	30.53205356

C	4.94144515	18.44390933	32.60283352
H	5.57229088	18.75121267	33.44948727
H	5.02844438	17.35882123	32.49432244
C	2.63511260	16.72528985	29.03580875
H	2.85207695	15.69626850	29.33145019
H	2.85141500	17.39537351	29.87476176
N	3.54419578	18.74902722	32.85015340
H	3.23238357	19.69544549	32.66122251
N	-0.63520210	20.63701972	27.53409834
H	-0.62586192	20.75466006	28.54252935
N	-0.00003684	24.83600442	32.16698287
H	1.01265959	24.79872907	32.09543345
C	9.40919620	21.24081292	29.68198286
O	10.30710437	21.23867690	28.86311663
C	9.92750627	19.52306547	31.44327414
O	8.71200603	18.77798131	31.28008116
C	10.51028436	19.28297744	32.80788251
C	6.29503043	21.68692892	27.72177232
S	5.33596927	23.00077126	28.49836253
H	10.60100709	19.12084091	30.67569857
H	11.49981713	19.74595943	32.91243084
H	9.86342788	19.70280931	33.59126338
H	10.61050503	18.20851298	32.99643201
H	8.04794398	19.20797926	31.84522394
H	5.94528729	20.70163936	28.05546911
H	6.11444446	21.73390330	26.64058562
C	7.79294172	21.78424298	27.94175638
H	8.15498691	22.77836028	27.64252152
H	8.31326631	21.05608085	27.30702139
C	9.70091220	21.00360880	31.14878911
H	8.89639022	21.38235932	31.79544810
H	10.61093948	21.56745187	31.38240012
N	8.14811994	21.51698894	29.32610169
H	7.40607976	21.54704245	30.02398578
H	7.40607976	21.54704245	30.02398578

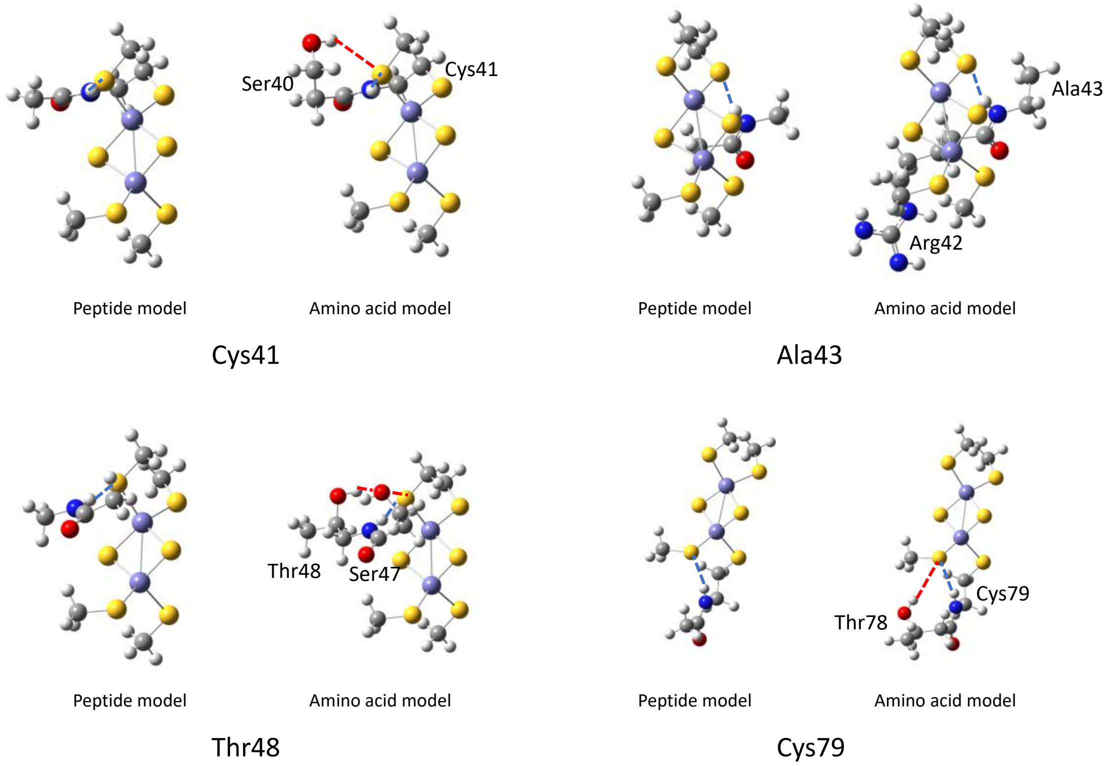

Figure S1. Illustration of the peptide and amino acid models using Cys41, Ala43, Thr48 and Cys79 ([Cys79+Thr78]).

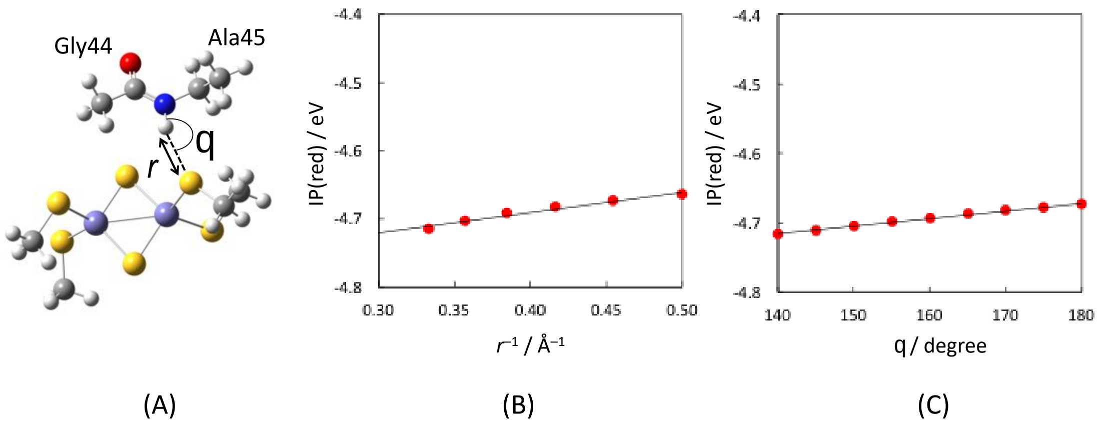

Figure S2. Change in *IP(red)* values by the structural change in the hydrogen bonds. (A) A model structure using Gly44 and Ala45. All internal structural parameters (bond distances, angles and dihedral angles) are fixed but only the $\boldsymbol{r}$ and θ are changed. The plots of *IP(red)* values against (B) inverse of the S-H distance ($\boldsymbol{r}^{-1}$) and (C) the N-H-S angle (θ).
